# Rare internal hernia following pancreatoduodenectomy: A case report

**DOI:** 10.1016/j.ijscr.2024.110673

**Published:** 2024-11-28

**Authors:** Teruyuki Tsujii, Kosei Takagi, Yasuo Nagai, Kazuya Yasui, Tomokazu Fuji, Toshiyoshi Fujiwara

**Affiliations:** Department of Gastroenterological Surgery, Okayama University Graduate School of Medicine, Dentistry, and Pharmaceutical Sciences, Okayama, Japan

**Keywords:** Pancreatoduodenectomy, Hernia, Abdominal

## Abstract

**Introduction:**

Pancreatoduodenectomy (PD) is a complex procedure with a high morbidity rate. Internal hernia following PD is a rare but potentially life-threatening complication. Herein, we describe a rare case of internal hernia after PD.

**Presentation of case:**

A 76-year-old man who underwent subtotal stomach-preserving PD 7 years ago presented with vomiting and abdominal pain. Abdominal computed tomography revealed an internal hernia. Because conservative treatment failed, surgical intervention was performed. Intraoperative findings revealed efferent loop herniation in the space between the afferent loop near the Braun anastomosis and transverse mesocolon. The hernia was repositioned and the mesenteric defect was closed.

**Discussion:**

This is an extremely rare case of an internal hernia that developed 7 years after PD. As conservative management provides a little chance for improvement, precise diagnosis and prompt re-intervention are essential for the management of internal hernia. In this case, the hernial orifice developed in the space between the afferent and efferent loops and the transverse mesocolon. Internal hernia could be a differential diagnosis in patients with ileus after PD.

**Conclusion:**

This study provided a detailed description of an extremely rare case of internal hernia following PD. Therefore, internal hernias should be considered in patients undergoing PD.

## Highlights

• Internal hernia following pancreatoduodenectomy (PD) is a rare.
• We present an extremely rare cause of internal hernia after PD.
• Precious diagnosis and prompt intervention are essential for management of internal hernia following PD.

## 1 Introduction

Pancreatoduodenectomy (PD) is a complicated procedure with a high morbidity incidence [1,2]. Among various complications after PD such as postoperative pancreatic fistula, bile leakage, and delayed gastric emptying, internal hernia is a rare but potentially life-threatening complication. Previous studies have reported the development of internal hernias into the Treitz fossa or Petersen's space after PD [[3], [4], [5]]. However, other sites of internal hernias could develop, including the Braun anastomosis. Herein, we describe a rare case of internal hernia after PD. This case report is in line with the SCARE Guidelines 2023 criteria [6].

## 2 Case presentation

A 76-year-old man underwent subtotal stomach-preserving PD 7 years ago for an intraductal papillary mucinous neoplasm. Reconstruction was performed using a modified version of the method described by Child, including hepaticojejunostomy, antecolic gastrojejunostomy, and Braun anastomosis [7].

During the follow-up, the patient was found to have no recurrence. However, the patient presented with vomiting and abdominal pain and was diagnosed with ileus at another institution. Initially, the patient was treated conservatively with nasogastric decompression, which did not improve his symptoms. Consequently, the patient was referred to our institution.

**Fig. 1:**
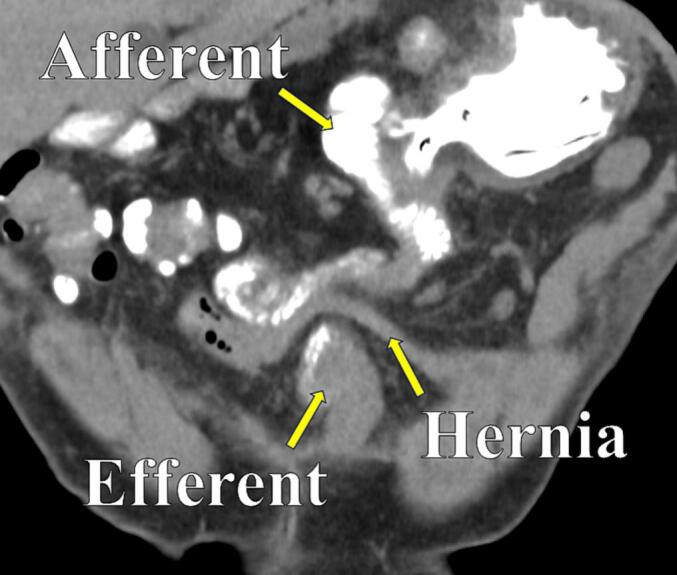
Abdominal computed tomography image revealing an internal hernia.

Laboratory tests revealed normal results with no elevated inflammatory response. Abdominal computed tomography and gastrointestinal fluoroscopy revealed the presence of an internal hernia (Fig. 1). Endoscopic intervention was attempted to treat the internal hernia. However, it failed due to jejunal obstruction on the anal side of the Braun anastomosis. Finally, a surgical intervention was performed.

**Fig. 2:**
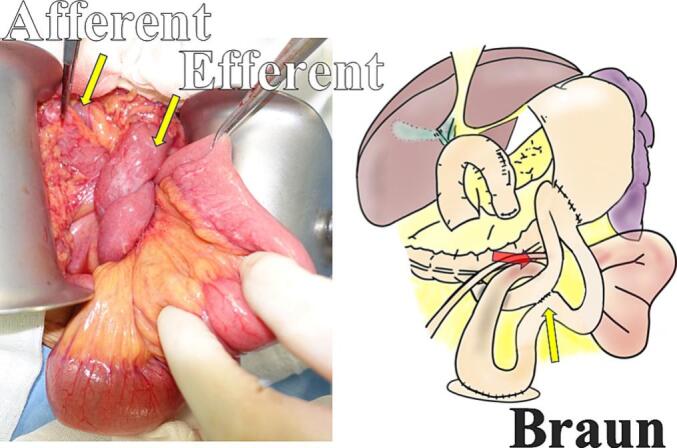
Intraoperative findings showing a herniation of the efferent loop in the space between the afferent loop near the Braun anastomosis and the transverse mesocolon (red arrow). (For interpretation of the references to colour in this figure legend, the reader is referred to the web version of this article.)

**Fig. 3:**
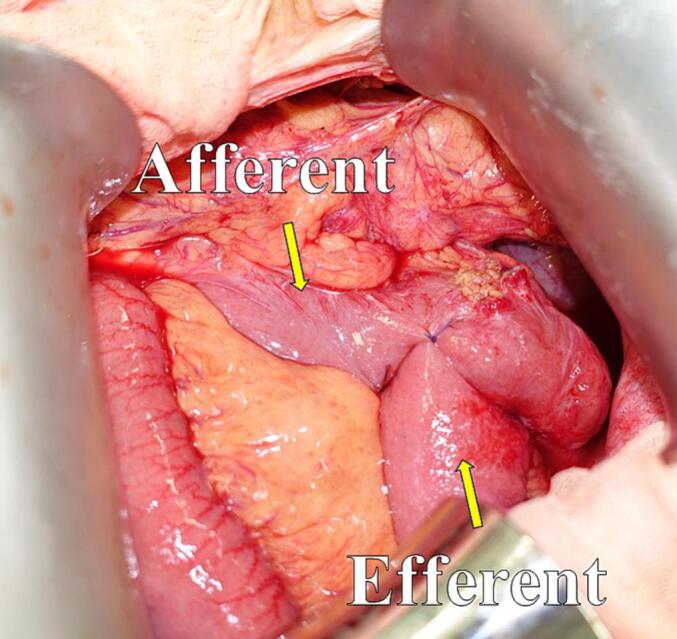
Intraoperative findings showing the repositioning of the herniation.

**Fig. 4:**
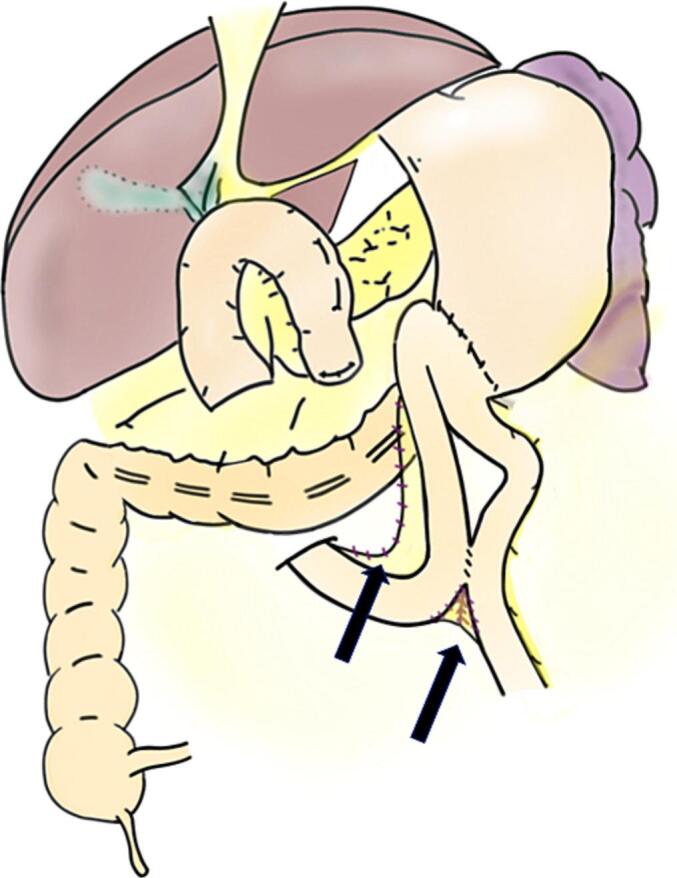
Shema following the closure of the mesenteric defect (black arrows).

Laparotomy was performed. Following dissection of intraabdominal adhesions, we found herniation of the efferent loop in the space between the afferent loop near the Braun anastomosis and transverse mesocolon (Fig. 2). The hernia was repositioned, and no bowel ischemia was observed (Fig. 3; Supporting Video 1). Finally, the mesenteric defect was closed (Fig. 4). The operative time was 80 min with no estimated blood loss.

The patient recovered from the ileus and was discharged 15 days postoperatively. During the 6-month follow-up period, the patient had no ileus recurrence.

## 3 Discussion

The present study presents an extremely rare case of an internal hernia that developed 7 years after PD. A precise diagnosis is important for developing a treatment strategy. In the present case, the patient was diagnosed with ileus and treated conservatively. Subsequently, surgical intervention was considered for the patient with a potential diagnosis of internal hernia. Conservative management of internal hernias provides little chance of improvement. Therefore, timely diagnosis and prompt reintervention are essential for managing internal hernias [5].

Internal hernia following PD is rare but occasionally occurs. To our knowledge, several studies have reported internal hernias following open PD [3,8] and minimally invasive PD [4,5,9]. Internal hernia has been reported to be more common in minimally invasive PD than in open PD due to fewer peritoneal adhesions [4,5]. Moreover, the sites of internal hernia are mostly the Treitz fossa or Petersen's space after PD [[3], [4], [5]]. Closure of the mesenteric defect is proposed to prevent the development of an internal hernia [4,5,9].

The potential pathogenesis of this case is extremely rare. First, an internal hernia developed after open PD. In open PD using a modification of the method described by Child, a large space is present between the afferent and efferent loops and the transverse mesocolon. Normally, this space is not closed during PD. In this case, adhesions occurred in the space, leading to the development of a hernial orifice. Regarding our surgical technique, our standard protocol included the Braun anastomosis to reduce the incidence of delayed gastric emptying and the intragastric bile reflux [10]. Finally, the efferent loop herniated into the hernial orifice, resulting in an internal hernia. Therefore, the prevention of this rare internal hernia was difficult. An important finding in this case was that an internal hernia was one of the differential diagnoses in a patient with ileus after PD.

## 4 Conclusion

The present study describes a rare case of internal hernia following PD that was successfully treated with surgical intervention by repositioning the hernia and closing the defect. Precise diagnosis and prompt intervention are essential for the management of internal hernias following PD.

The following is the supplementary data related to this article.

Video 1 Rare internal hernia following pancreatoduodenectomy. Video 1

## Consent

Written informed consent was obtained from the patient for publication and any accompanying images. A copy of the written consent is available for review by the Editor-in-Chief of this journal on request.

## Ethical approval

We certify that this kind of manuscript does not require ethical approval (exemption) by the Ethical Committee of our institution.

## Guarantor

Kosei Takagi.

## Research registration number

Not applicable.

## Funding

There are no sources of funding.

## Author contribution

Teruyuki Tsujii: Conceptualization, Patient management.

Kosei Takagi: Data curation, Writing- Original draft preparation.

Yasuo Nagai: Conceptualization, Patient management.

Kazuya Yasui: Conceptualization, Patient management.

Tomokazu Fuji: Patient management, Writing- Reviewing and Editing.

Toshiyoshi Fujiwara: Supervision, Writing- Reviewing and Editing.

## Declaration of competing interest

The authors have no conflicts of interest to declare.

## References

[1] Aoki S., Miyata H., Konno H., Gotoh M., Motoi F., Kumamaru H. (2017). Risk factors of serious postoperative complications after pancreaticoduodenectomy and risk calculators for predicting postoperative complications: a nationwide study of 17,564 patients in Japan. J. Hepatobiliary Pancreat. Sci..

[2] Takagi K., Yagi T., Yoshida R., Shinoura S., Umeda Y., Nobuoka D. (2016). Surgical outcome of patients undergoing pancreaticoduodenectomy: analysis of a 17-year experience at a single center. Acta Med. Okayama.

[3] Yamanaka T., Araki K., Hagiwara K., Ishii N., Tsukagoshi M., Igarashi T. (2017). Internal hernia into the treitz fossa after pancreaticoduodenectomy. Case Rep. Gastroenterol..

[4] Brinkman D.J., Van Hilst J., Luyer M.D. (2020). Internal herniation following laparoscopic pancreatoduodenectomy. BMJ Case Rep..

[5] Viti V., Ginesini M., Ripolli A., Boggi U. (2024). Internal hernia through the Treitz fossa after robotic pancreatoduodenectomy: pathogenesis and preventive measures. Updat. Surg..

[6] Sohrabi C., Mathew G., Maria N., Kerwan A., Franchi T., Agha R.A. (2023). The SCARE 2023 guideline: updating consensus Surgical CAse REport (SCARE) guidelines. Int. J. Surg..

[7] Takagi K., Yoshida R., Yagi T., Umeda Y., Nobuoka D., Kuise T. (2017). Radiographic sarcopenia predicts postoperative infectious complications in patients undergoing pancreaticoduodenectomy. BMC Surg..

[8] Patel P., Patel N., Atia A., Murthy R., Young M. (2013). Massive gastric dilatation secondary to internal hernia obstructing the biliary intestinal limb of whipple procedure. ACG Case Rep J..

[9] Qin K., Wu Z., Jin J., Shen B., Peng C. (2018). Internal hernia following robotic assisted pancreaticoduodenectomy. Med. Sci. Monit..

[10] Zhou Y., Hu B., Wei K., Si X. (2018). Braun anastomosis lowers the incidence of delayed gastric emptying following pancreaticoduodenectomy: a meta-analysis. BMC Gastroenterol..

